# Interpretable machine learning identifies metabolites associated with glomerular filtration rate in type 2 diabetes patients

**DOI:** 10.3389/fendo.2024.1279034

**Published:** 2024-06-10

**Authors:** Tian-Feng An, Zhi-Peng Zhang, Jun-Tang Xue, Wei-Ming Luo, Yang Li, Zhong-Ze Fang, Guo-Wei Zong

**Affiliations:** ^1^ Department of Toxicology and Health Inspection and Quarantine, School of Public Health, Tianjin Medical University, Tianjin, China; ^2^ Department of Surgery, Peking University Third Hospital, Beijing, China; ^3^ Tianjin Key Laboratory of Environment, Nutrition and Public Health, Tianjin, China; ^4^ Department of Mathematics, School of Public Health, Tianjin Medical University, Tianjin, China

**Keywords:** type 2 diabetes, metabolomics, amino acids, acylcarnitine, machine learning, glomerular filtration rate, renal function

## Abstract

**Objective:**

The co-occurrence of kidney disease in patients with type 2 diabetes (T2D) is a major public health challenge. Although early detection and intervention can prevent or slow down the progression, the commonly used estimated glomerular filtration rate (eGFR) based on serum creatinine may be influenced by factors unrelated to kidney function. Therefore, there is a need to identify novel biomarkers that can more accurately assess renal function in T2D patients. In this study, we employed an interpretable machine-learning framework to identify plasma metabolomic features associated with GFR in T2D patients.

**Methods:**

We retrieved 1626 patients with type 2 diabetes (T2D) in Liaoning Medical University First Affiliated Hospital (LMUFAH) as a development cohort and 716 T2D patients in Second Affiliated Hospital of Dalian Medical University (SAHDMU) as an external validation cohort. The metabolite features were screened by the orthogonal partial least squares discriminant analysis (OPLS-DA). We compared machine learning prediction methods, including logistic regression (LR), support vector machine (SVM), random forest (RF), and eXtreme Gradient Boosting (XGBoost). The Shapley Additive exPlanations (SHAP) were used to explain the optimal model.

**Results:**

For T2D patients, compared with the normal or elevated eGFR group, glutarylcarnitine (C5DC) and decanoylcarnitine (C10) were significantly elevated in GFR mild reduction group, and citrulline and 9 acylcarnitines were also elevated significantly (FDR<0.05, FC > 1.2 and VIP > 1) in moderate or severe reduction group. The XGBoost model with metabolites had the best performance: in the internal validate dataset (AUROC=0.90, AUPRC=0.65, BS=0.064) and external validate cohort (AUROC=0.970, AUPRC=0.857, BS=0.046). Through the SHAP method, we found that C5DC higher than 0.1μmol/L, Cit higher than 26 μmol/L, triglyceride higher than 2 mmol/L, age greater than 65 years old, and duration of T2D more than 10 years were associated with reduced GFR.

**Conclusion:**

Elevated plasma levels of citrulline and a panel of acylcarnitines were associated with reduced GFR in T2D patients, independent of other conventional risk factors.

## 1 Introduction

Type 2 diabetes (T2D) has emerged as a major global health concern (1). According to the US Renal Data System, certain countries have reported high incidence rates of end-stage renal disease (ESRD) caused by diabetes, accounting for approximately 50% of cases (2, 3). Despite glucose control, the progression from T2D to diabetic nephropathy and ESRD is often inevitable (4). Glomerular filtration rate (GFR) is an independent predictor of the incidence of ESRD (5), which is recommended by the American Kidney Foundation as the most important basis for the definition, staging, screening, and monitoring of chronic kidney disease (CKD) (6). However, the commonly used creatinine-based eGFR is not sensitive to detect incipient kidney dysfunction (7). Therefore, it is necessary to identify novel biomarkers for early detection of the onset and progression of renal function.

With advances in metabolomics technology, it has become feasible to identify novel makers that can predict disease (8, 9). Numerous studies have demonstrated significant metabolic disorders associated with diabetes and diabetes-related complications (10, 11). Cross-sectional studies have shown that the plasma amino acids related to the urea cycle (including ornithine and citrulline) and tryptophan, as well as most short- and medium-chain acylcarnitines, are associated with CKD (12, 13). Additionally, an animal experiment showed that amino acid administration can increase eGFR (14). However, there is limited data linking the metabolome to the development of DKD. In this study, we aimed to identify GFR-associated metabolic phenotypes in patients with T2D, which may serve as novel biomarkers for kidney function and the pathophysiology of CKD in T2D patients.

Due to the multidimensional and highly correlated nature of metabolomics data, it is necessary to employ appropriate methods to effectively narrow down the range of significant candidate biomarkers. This is essential to achieve higher learning speed, improved generalization ability, and enhanced interpretability of classification models (15–17). Orthogonal partial least squares discriminant analysis (OPLS-DA) is considered a powerful statistical analysis tool for addressing collinearity and information redundancy issues (18, 19). Machine learning (ML) algorithms provide new techniques for integrating and analyzing various omics data, aiding in the discovery of new biomarkers and extensively used in disease prediction (20). For example, metabolomic-based predict individual multi-disease outcomes (21); metabolic detection of malignant brain gliomas through support vector machine-based machine learning (22); a novel deep convolution neural network-based brain tumor classification model (23); brain tumor identification using data augmentation and transfer learning approach (24); U-Net-Based models towards optimal MR brain image segmentation (25); an intuitionistic approach for the predictability of anti−angiogenic inhibitors in cancer diagnosis (26).

In this study, the combination of plasma metabolomic profiling and ML approaches may lead to the identification of metabolic profiles that enhance our understanding of the underlying causes of renal impairment.

## 2 Materials and methods

### 2.1 Study settings and subjects

From May 2015 to August 2016, a total of 4352 consecutive patients with T2D were enrolled at Liaoning Medical University First Affiliated Hospital (LMUFAH), Jinzhou, China. T2D was diagnosed by the 1999 WHO’s criteria (27) or treated with antidiabetic drugs. Inclusion criteria for this study were: 1) Patients diagnosed as T2D or treated with antihyperglycemic therapy; 2) Complete eGFR, amino acid, and Acylcarnitine. Exclusion criteria were: 1) T2D patients under 18 years old; 2) Patients with cancer. A total of 1626 subjects were included.

From April 2018 to April 2019, a total of 1011 consecutive patients with T2D were enrolled at the Second Affiliated Hospital of Dalian Medical University (SAHDMU). Removing 295 samples of missing data, 716 patients with complete data were included as an external validation cohort.

The Ethics Committee for Clinical Research of LMUFAH and SAHDMU approved the ethics of the study, and informed consent was waived due to the retrospective character of the cross-sectional study, which is consistent with the Helsinki Declaration.

### 2.2 Date collection and clinical definitions

We retrieved the data from electronic medical records including demographic and anthropometric information, current clinical data, and diabetes course. Demographic data included gender, age, smoking, and drinking. Anthropometric measurements included weight, height, systolic blood pressure (SBP), and diastolic blood pressure (DBP). Clinical parameters encompassed plasma creatinine (SCR), cholesterol (CHOL), triglycerides (TG), high-density lipoprotein cholesterol (HDL-C), and low-density lipoprotein cholesterol (LDL-C). Medication information included antidiabetic agents and lipid-lowering drugs. Amino acids and acylcarnitines in plasma were quantified using liquid chromatography coupled with mass spectrometry.

According to the eGFR: Improving Global Outcomes (KDIGO) Diabetes Work Group (28), three patient groups were enrolled: normal or elevated (NOE) eGFR (eGFR≥90mL/min•1.73m^2^), mild reduction (MR) eGFR (60≤eGFR<90mL/min•1.73m^2^) and moderate or severe reduction (MOMR) eGFR (eGFR<60mL/min•1.73m^2^). CKD-EPI formula (29): Calculation formula:

1. Female: ① SCR ≤ 0.7 mg/dl, GFR = 144*(SCR/0.7)−0.329*(0.993)^age^; ② SCR > 0.7 mg/dl, GFR = 144*(SCR/0.7)−1.209*(0.993)^age^.
2. Male: ① SCR ≤ 0.9 mg/dl, GFR = 141*(SCR/0.9)−0.411*(0.993)^age^; ② SCR > 0.9 mg/dl, GFR = 141*(SCR/0.9)−1.209*(0.993)^age^.

### 2.3 Determination of amino acids and acylcarnitine

The quantification of amino acids and acylcarnitines was conducted following the method described in previous studies (30). In brief, metabolomics analysis was performed using mass spectrometry. After fasting for a minimum of 8 hours, capillary whole blood samples were collected from the subjects and prepared as dry blood spots for metabolomic analysis. Metabolites were measured using direct infusion MS technology with the AB Sciex 4000 QTrap system (AB Sciex, Framingham, MA, USA). High-purity water and acetonitrile from Thermo Fisher (Waltham, MA, USA) were used as the diluting agent and mobile phase, respectively. Amino acid and acylcarnitine quantification utilized isotope-labeled internal standards from Cambridge Isotope Laboratories (Tewksbury, Massachusetts, USA).

### 2.4 Statistical description

Continuous variables that followed a normal distribution were described as mean ± standard deviation (SD), while non-normally distributed variables were presented as median (interquartile range). Categorical data were reported as numbers (percentages). To test for differences among different eGFR groups, we employed chi-square tests for categorical variables, one-way ANOVA for variables with a normal distribution, and the Kruskal-Wallis H test for variables with a skewed distribution.

### 2.5 Data preprocessing and data set division

Variables with more than 20% missing data were excluded from the analysis. The missing values were then interpolated using the multiple imputation (MI) method (31), which is an advanced technique for handling missing data. Subsequently, the dataset was randomly divided into a training set (70%) and an internal test set (30%).

### 2.6 Feature selection

The objective of feature selection is to eliminate redundant factors, reduce the complexity of the prediction model, and improve accuracy without losing key information. Regarding general information and physical indicators, we applied zero-mean normalization (Z-Score) to numerical variables and performed feature selection using the least absolute shrinkage and selection operator (LASSO). LASSO regression includes a regularization/penalty term in the cost function to prevent overfitting and ensure that the model selects relevant features while disregarding correlated ones. According to the one standard error rule (1SE rule), the optimal value corresponds to the simplest model, with the cross-validation error no more than one standard error above the minimum (32). For plasma amino acids and acylcarnitines, we first applied the Benjamini-Hochberg false discovery rate (FDR) procedure for multiple test adjustments. Additionally, we calculated the fold change (FC) of metabolites between different groups. The FC represents the difference in expression levels of a particular metabolite between two groups based on quantitative results. Furthermore, after logarithmic transformation of the variables, multivariate analysis was performed using the OPLS-DA method. Metabolites with FDR < 0.05, FC > 1.2, and VIP > 1 were considered significant.

### 2.7 Model development and validation

Models can be implemented in Python 3.9 using standard libraries that are publicly available, including pandas (1.5.3), numpy (1.23.5), scikit-learn (1.2.1), and matplotlib (3.7.0). We constructed four predictive models: conventional logistic regression model (LR), support vector machine (SVM), random forest (RF), and eXtreme Gradient Boosting (XGBoost). Four models were first trained on the randomly selected training set (fivefold-stratified cross-validation) and then applied to the withheld test set to access the final performance. We employed a grid search method to select the optimal hyperparameters for RF (n_ estimators, max_depth, min_samples_split, and min_samples_leaf) and XGBoost (n_ estimators, max_depth, min_child_weight, gamma, and subsample) models. For the LR and SVM models, we implemented the default settings provided by scikit-learn. AUROC is a widely used metric for evaluating the performance of classification models, which provides a comprehensive assessment of the model’s sensitivity and specificity trade-off at different thresholds. The range of AUC values is typically explained from 0.5 to 1, with a higher value indicating a better ability to distinguish between different classes of samples. AUPR, as a complimentary assessment, considers the trade-offs between precision (or positive predictive value) and recall (or sensitivity) and it is more robust for imbalanced datasets. The AUPRC ranges from 0 to 1 with a value of 0 signifies no positive examples identified and a value of 1 indicating perfect identification of all positive examples. In addition, the calibration plot and Brier score (BS) were used to evaluate calibration.

### 2.8 Model interpretation

We used Shapley Additive exPlanations (SHAP) to unlock the machine learning results (33, 34). SHAP measured the impact of genus characteristics on predicted scores by employing a game theory approach based on test sets, which allowed us to assess the importance of each feature. The SHAP value, which quantifies the influence of a variable on a prediction in terms of direction and magnitude, was computed by considering the prediction outcome for every possible combination of features. This comprehensive analysis provided valuable information about the contribution of each genus characteristic to the overall predictions.

Statistical description and feature selection were conducted using R V4.2.2, which is widely recognized as a popular statistical analysis software. For building the model, Python v3.9.13 was chosen due to its extensive machine-learning libraries and tools, such as scikit-learn, which offer a wide range of tuning parameters and algorithm options.

## 3 Results

### 3.1 Baseline characteristics

**Figure 1:**
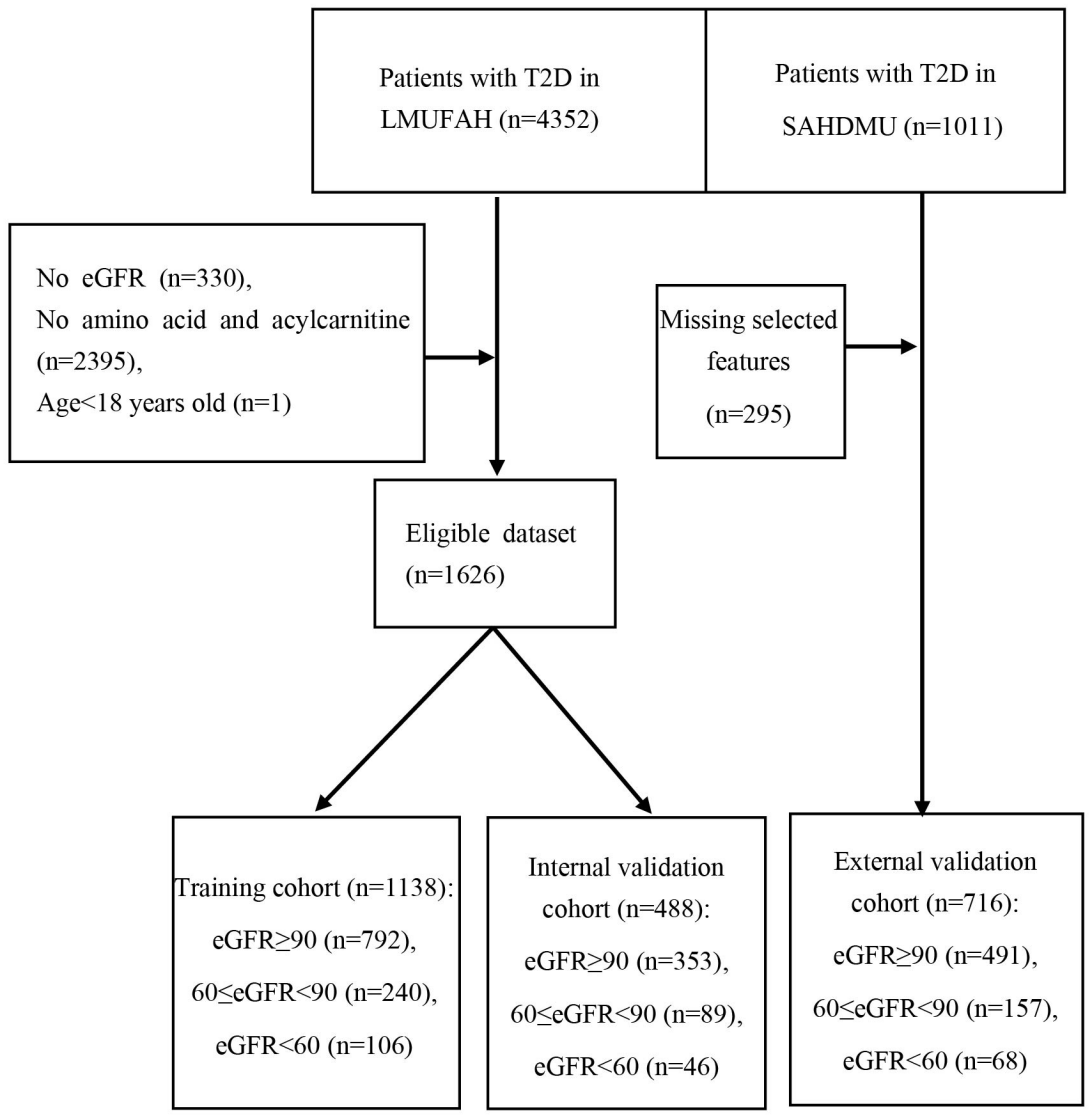
Schematic diagram of subject screening process.

**Table 1** presents the patient characteristics of both LMUFAH and SAHDMU. LMUFAH included a total of 1626 patients, while SAHDMU consisted of 716 patients with type 2 diabetes (T2D). Among the 1626 T2D patients in LMUFAH, 1145 (70.4%) had an estimated glomerular filtration rate (eGFR) ≥ 90 mL/min•1.73m², 329 (20.2%) had 60 ≤ eGFR < 90 mL/min•1.73m², and 68 (9.4%) had an eGFR < 60 mL/min•1.73m². The subject selection procedure is depicted in **Figure 1**.

**Table 1:** Patient characteristics.

eGFR		LMUFAH			p	SAHDMU			p
		NOE	MR	MOSR		NOE	MR	MOSR	
**n**		1145	329	152		491	157	68	
**gender (%)**	man	548 (47.9)	149 (45.3)	77 (50.7)	0.521	252 (51.3)	85 (54.1)	31 (45.6)	0.498
	female	597 (52.1)	180 (54.7)	75 (49.3)		239 (48.7)	72 (45.9)	37 (54.4)	
**Current smoking**		837 (73.1)	267 (81.2)	113 (74.3)	0.012	389 (79.2)	138 (87.9)	58 (85.3)	0.036
**Current drinking**		908 (79.3)	281 (85.4)	125 (82.2)	0.041	440 (89.6)	145 (92.4)	63 (92.6)	0.486
**Age, years**		54.92 (12.57)	68.36 (10.36)	65.66 (12.10)	<0.001	56.15 (12.21)	69.30 (9.86)	66.79 (9.76)	<0.001
**BMI**		25.95 (4.03)	25.59 (3.32)	25.79 (3.82)	0.339	26.71 (4.12)	26.03 (3.05)	27.42 (3.32)	0.032
**Duration of T2D, years**		7.40 (7.40)	10.74 (9.09)	14.03 (8.82)	<0.001	8.80 (7.87)	12.61 (9.77)	17.37 (9.36)	<0.001
**SBP**		141.79 (22.69)	149.01 (21.97)	151.62 (25.31)	<0.001	145.50 (20.90)	152.78 (19.42)	157.19 (21.37)	<0.001
**DBP**		82.94 (12.59)	80.33 (13.31)	80.05 (14.51)	0.001	82.65 (11.97)	78.18 (11.95)	80.74 (12.92)	<0.001
**Total cholesterol, (mmol/l)**		4.94 (1.32)	4.76 (1.42)	4.99 (1.61)	0.109	5.04 (1.28)	4.85 (1.31)	5.51 (1.71)	0.003
**HDL_C**		1.14 (0.35)	1.15 (0.33)	1.12 (0.35)	0.763	1.18 (0.34)	1.20 (0.31)	1.23 (0.31)	0.497
**LDL_C**		2.75 (0.93)	2.66 (1.02)	2.55 (0.91)	0.044	2.55 (0.83)	2.50 (0.87)	2.51 (0.92)	0.805
**Triglyceride, (mmol/L)**		1.64 (1.13-2.41)	1.50(1.06-2.15)	1.86(1.17-2.81)	0.003	1.60(1.11-2.24)	1.44(1.05-1.95)	2.04(1.31-3.23)	<0.001
**Antidiabetic agents (%)**		512 (81.1)	133 (77.8)	67 (80.7)	0.615	435 (88.6)	142 (90.4)	61 (89.7)	0.614
**Lipid-lowering agents (%)**		512 (81.1)	126 (73.7)	70 (84.3)	0.056	295(100)	0	46(100)	–

NOE, Normal or elevated eGFR; MR, Mild reduction eGFR; MOMR, Moderate or severe reduction eGFR; SBP, Systolic blood pressure, mmHg; DBP, Diastolic blood pressure, mmHg; HDL_C, High-density lipoprotein cholesterol, mmol/L; LDL_C, Low-density lipoprotein cholesterol, mmol/L.

Data are mean (standard deviation), median (IQR), or n (%).

### 3.2 Feature selection

#### 3.2.1 Pairwise comparison of clinical factors

After excluding variables with more than 20% missing data, Lasso was used to screen variables for 12 clinical factors. Eight variables in the NOE vs MR group and seven variables in the NOE vs MOMR group were selected to be included in the model (**Supplementary Figure 1**). Among these variables, age, SBP, TG, HDL_C, and gender were all included.

#### 3.2.2 Pairwise comparison of differentially expressed metabolites in plasma

In metabonomic analysis, we obtained adjusted p-value (FDR), FC, and VIP for every metabolite (**Supplementary Table 1**). Compared with NOE, four AAs (asparagine, citrulline, leucine and valine) and six AcylCNs (3-hydroxyisovalerylcarnitine (C5-OH), glutarylcarnitine (C5DC), octanoylcarnitine (C8), decanoylcarnitine (C10), lauroylcarnitine (C12), and tetradecenoylcarnitine (C14:1) were elevated in the other two groups and had significant differences (FDR<0.05) (**Supplementary Table 1**). A volcano map based on FC and p values is displayed in **Supplementary Figure 2**, with over-expressed and under-expressed metabolites marked in red and blue colors, respectively. OPLS-DA analysis, a widely used multivariate analysis method in metabolomics, was employed to identify significant metabolites for predicting sample classes. We used this method to screen for important metabolites with VIP > 1 (**Supplementary Figure 3**). In the NOE vs MR group, two metabolites (C5DC and C10) were selected for the final model. In the NOE vs MOMR group, citrulline and nine acylcarnitines met the inclusion criteria.

### 3.3 Model performance

We developed machine learning prediction methods, including LR, SVM, RF, and XGBoost. Model 1 utilized common clinical factors, while Model 2 integrated plasma metabolites and clinical factors. Due to the data imbalance, we employed the area under the precision-recall curve (AUPRC) as the primary evaluation metric and the area under the receiver operating characteristic curve (AUROC) as the secondary evaluation metric.

#### 3.3.1 NOE vs MR

In the internal validation cohort, the XGBoost model incorporating clinical factors demonstrated the best predictive performance [AUPRC: 0.561 (0.45-0.66), AUROC: 0.799 (0.74-0.83)]. However, there were no significant differences observed when compared to the other models (P > 0.05) (**Supplementary Table 2**, **Supplementary Figure 4**, **Table 2**).

**Table 2:** Comparison of performance of XGBoost models in the internal validation cohort.

	NOE vs M-GFE		P	NOE vs MOMR		P
	XGBoost1	XGBoost2		XGBoost1	XGBoost2	
AUROC	0.799(0.74-0.85)	0.784(0.73-0.84)	0.27	0.794(0.73-0.85)	0.894(0.85-0.94)	<0.001
AUPRC	0.561(0.45-0.66)	0.544(0.43-0.65)	0.20	0.374(0.24-0.53)	0.648(0.50-0.77)	<0.001

XGBoost, extreme Gradient Boosting.

XGBoost1 is a model that only includes traditional clinical factors; XGBoost2 adds plasma metabolites.

P, Delong test for the area under the curve of the receiver operating characteristic curve and precision-recall curve.

NOE, Normal or elevated eGFR; MR, Mild reduction eGFR; MOMR, moderate or severe reduction eGFR.

#### 3.3.2 NOE vs MOMR

The predictive abilities of all models using only clinical factors were not high, while the addition of metabolite features significantly promoted the prediction ability of renal function (**Supplementary Table 3**, **Supplementary Figure 5**). The performance of the XGBoost2 model was optimal in the internal validation cohort [AUPRC: 0.648 (0.50-0.77), AUROC: 0.894 (0.85-0.94)] compared with LR2, AUPRC and AUROC increased by 23% and 6.9%, respectively (**Supplementary Table 3**, **Table 2**). At the optimal threshold determined by Youden’s index, we obtained the precision, recall, and false negative rate in the internal validation cohort. Among them, the XGBoost2 model had the highest precision (0.532), while the SVM model had the best recall (0.804) and false negative rate (0.196) (**Table 3**). The result of the calibration evaluation is shown in **Supplementary Figure 6**. Among the four models, the XGBoost2 model had the best consistency with the true situation and with the smallest BS score (0.064).

**Table 3:** The performance of the four models for NOE vs MOMR after the selected thresholds in the internal validation cohort.

model	Cutoff	Precision	Recall	False Negative Rate
LR2	0.143	0.333	0.717	0.283
SVM2	0.101	0.272	0.804	0.196
RF2	0.240	0.507	0.695	0.304
XGBoost2	0.080	0.532	0.717	0.283

LR, logistic regression; SVM, support vector machine; RF, random forest; XGBoost, extreme Gradient Boosting.

### 3.4 GFR influencing factors assessment

We selected the optimal XGBoost models to further analyze the influence of predictors on reduced GFR.

#### 3.4.1 NOE vs MOMR

We interpret the model with metabolites using a SHAP plot and find that age older than 65 and C5DC higher than 0.08μmol/L are important predictors of mild reduction GFR (**Supplementary Figure 7**).

#### 3.4.2 NOE vs MOMR

**Figure 2:**
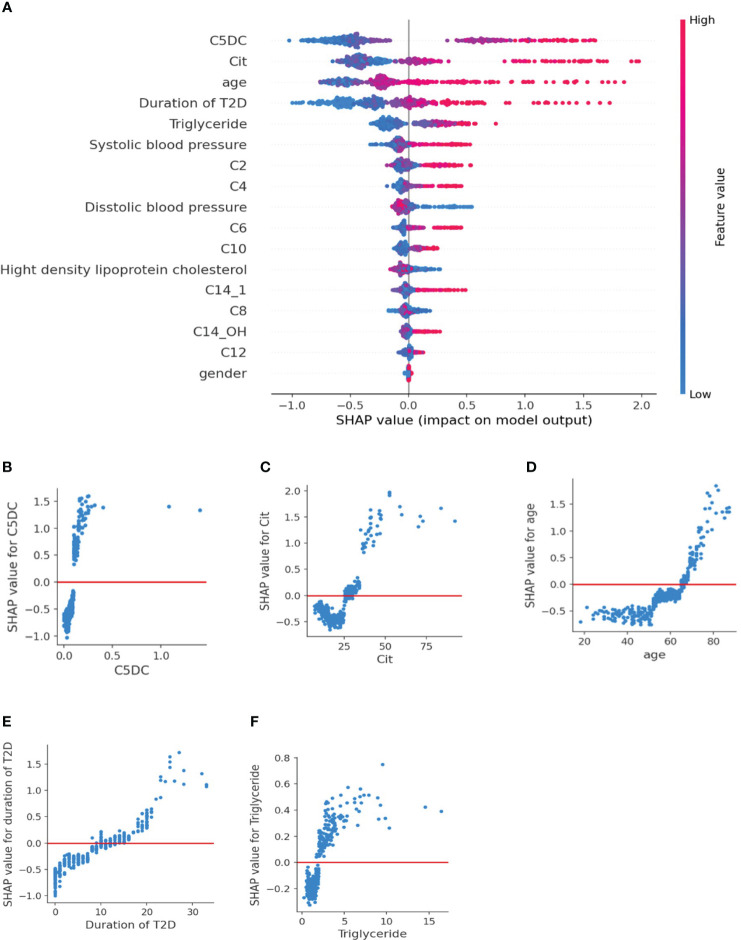
SHAP plot of XGBoost2 model for NOE vs MOMR. **(A)** SHAP summary plot. Features are ranked from top to bottom according to their importance. Each dot on the plot is a SHAP value for each feature. Red dots indicate high feature values, but blue dots represent low feature values for the per-patient model. **(B–F)** SHAP dependence plot. The SHAP value of each feature exceeded zero, indicating an increased risk of moderate or severe reduction of eGFR. C5DC, Cit, age, duration of T2D, and triglyceride were risk factors for moderate or severe reduction of eGFR. SHAP, Shapley Additive explanation; C2, acetylcarnitine; C4, butyrylcarnitine; C6, hexanoylcarnitine; C8, octanoylcarnitine; C10, decanoylcarnitine; C12, lauroylcarnitine; C14:1, tetradecenoylcarnitine; C14-OH, 3-hydroxyl-tetradecanoylcarnitine.

We constructed a SHAP summary plot to assess the importance of features in the XGBoost2 mode (**Figure 2A**). As shown in the SHAP summary plot, the red dots indicate high feature values, however, blue dots represent low feature values. The higher the SHAP value, the greater the risk of moderate or severe reduction of GFR. C5DC, age, citrulline, and duration of T2D contributed more to the model and they are all the risk factors for moderate or severe reduction of GFR. The SHAP dependence plot more clearly shows the effect of a single indicator on the outcome of the study. (**Figures 2B–F**). When the SHAP value of each characteristic exceeds zero, it indicates an increased risk of moderate or severe reduction GFR. C5DC higher than 0.1μmol/L, Cit higher than 26μmol/L, age greater than 65 years old, duration of T2D more than 10 years, and triglyceride higher than 2 mmol/L were associated with increased risk of moderate or severe reduction GFR.

### 3.5 External validation

#### 3.5.1 Selection of models for external validation

The comparison between the two groups demonstrated that the XGBoost model exhibited superior performance, thus we selected this model for external validation. We proceeded to assess the risk of reduced GFR in patients with type 2 diabetes (T2D) with and without metabolite features.

#### 3.5.2 Characteristics of the external validation cohort

Of the 716 patients with T2D, 491 (68.5%) had eGFR≥90mL/min•1.73m^2^; 157 (22%) had 60≤eGFR<90mL/min•1.73m^2^; 68 (9.5%) had eGFR<60mL/min•1.73m^2^) (**Table 1**).

#### 3.5.3 Performance of external validation

**Figure 3:**
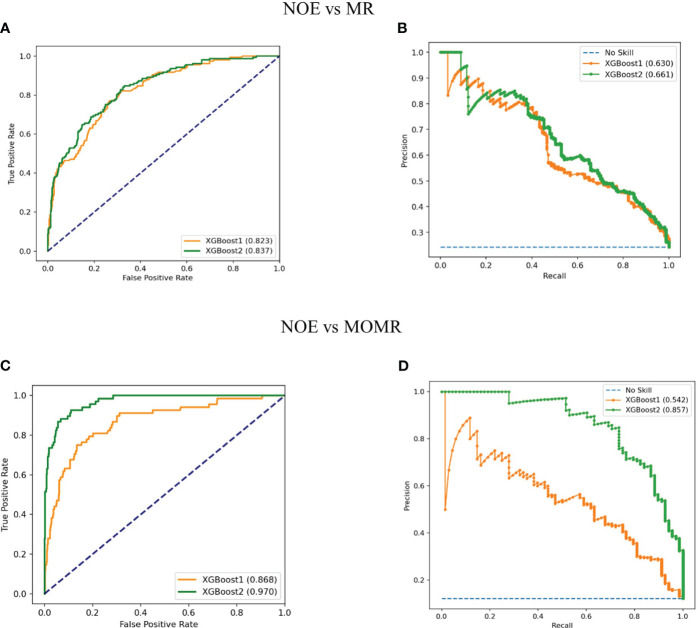
Performance of XGBoost models in the external validation cohort. **(A, C)** Receiver operating characteristic curves of models with clinical factors and the combination of plasma metabolites and clinical factors, respectively; **(B, D)** Precision recall curves of models with clinical factors and the combination of plasma metabolites and clinical factors, respectively. XGBoost1 is a model that only includes traditional clinical factors; XGBoost2 adds plasma metabolites. LR, logistic regression; SVM, support vector machine; RF, random forest; XGBoost, extreme Gradient Boosting; No skill is the reference line. NOE, Normal or elevated eGFR; MR, Mild reduction eGFR; MOMR, moderate or severe reduction eGFR.

**Figure 4:**
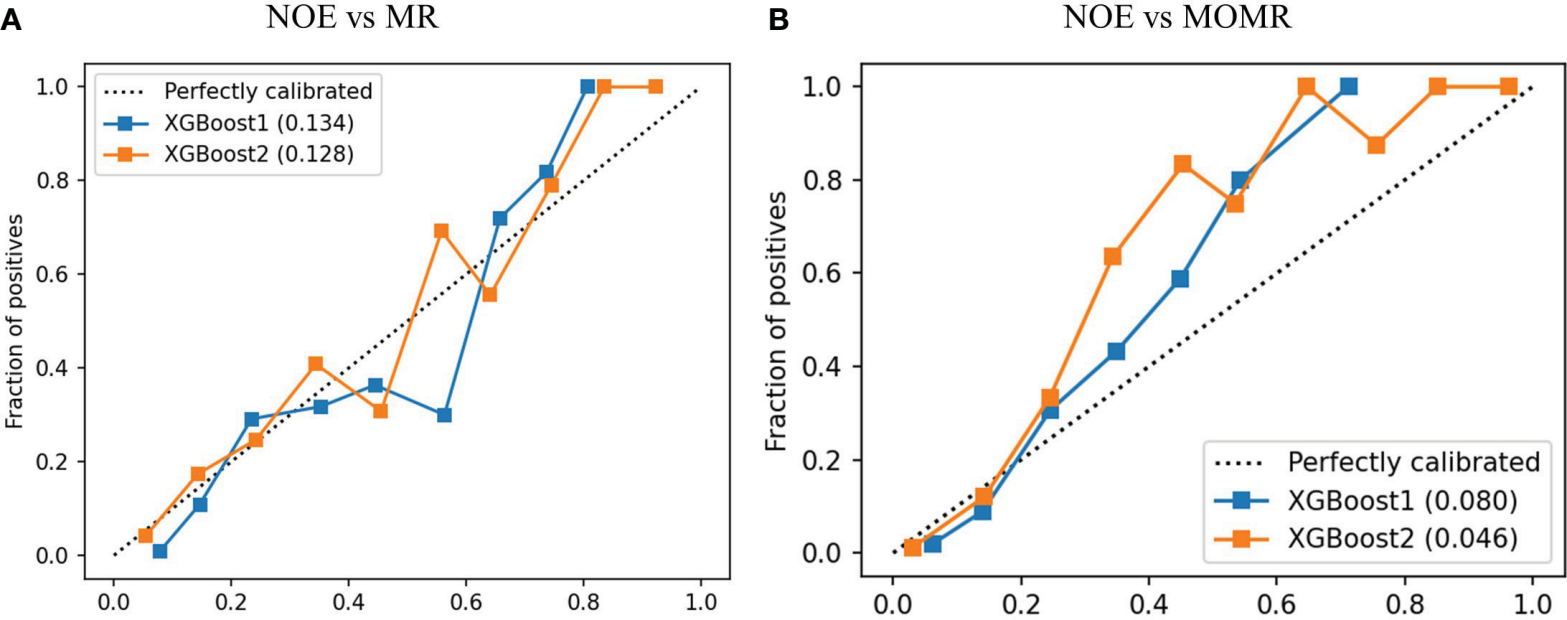
The calibration curve of XGBoost models in the external validation cohort. **(A)** was a comparison of NOE vs MR and **(B)** was a comparison of NOE vs MOMR. XGBoost1 is a model that only includes traditional clinical factors; XGBoost2 adds plasma metabolites. The values in brackets represent the Brier score of the corresponding prediction model. Perfectly calibrated is the reference line; XGBoost, extreme Gradient Boosting. NOE, Normal or elevated eGFR; MR, Mild reduction eGFR; MOMR, moderate or severe reduction eGFR.

The Discriminatory ability of XGBoost models was significantly promoted with the addition of plasma metabolites. XGBoost2 models with plasma metabolites and clinical factors performed best for NOE vs MR [AUPRC: 0.661(0.58-0.73), AUROC: 0.837 (0.80-0.88)] (**Table 4**, **Figure 3**) and for NOE vs MOMR [AUPRC: 0.857 (0.77-0.92), AUROC: 0.970 (0.95-0.98)] (**Table 4**, **Figure 3**). XGBoost2 models had the best consistency with the true situation and BS were both the smallest for NOE vs MR (0.128) and for NOE vs MOMR (0.046) (**Figure 4**).

**Table 4:** Comparison of performance of XGBoost models in the external validation cohort.

	NOE vs C-GFE		P	NOE vs MOMR		P
	XGBoost1	XGBoost2		XGBoost1	XGBoost2	
**AUROC**	0.823(0.79-0.86)	0.837(0.80-0.88)	0.10	0.868(0.82-0.91)	0.970(0.95-0.98)	<0.001
**AUPRC**	0.630(0.55-0.71)	0.661(0.58-0.73)	<0.001	0.542(0.42-0.67)	0.857(0.77-0.92)	<0.001

XGBoost, Extreme Gradient Boosting; AUROC, the area under the curve of the receiver operating characteristic curve; AUPRC, area under the precision recall curve; Ref, reference.

XGBoost1 is a model that only includes traditional clinical features; XGBoost2 adds plasma metabolite features.

P, Delong test for the area under the curve of the receiver operating characteristic curve and precision-recall curve.

NOE, Normal or elevated eGFR; MR, Mild reduction eGFR; MOMR, moderate or severe reduction eGFR.

## 4 Discussion

In contrast to previous studies, we employed three criteria to screen for important metabolic information: FDR < 0.05, FC > 1.2, and VIP > 1. As an extension of PLS-DA, OPLS-DA is capable of reducing model complexity and enhancing model interpretability without compromising predictive performance. This allows us to gain maximum insight into the differences between groups. Generally, the VIP value associated with a variable indicates its importance in explaining the X dataset and its association with the Y dataset. A VIP value greater than 1 indicates the variable’s significance in the analysis.

As the end products of cellular regulatory processes, metabolites are considered to be the ultimate response of biological systems to pathophysiological changes in various metabolic disorders. They closely reflect the disease phenotype and address a critical clinical need, as they represent the downstream expression of the genome, transcriptome, and proteome (35). The focus of the study was on profiling the continuously changing metabolites from normal or elevated eGFR to mild reduction eGFR, and then to moderate or severe reduction eGFR. In the cohort of patients with T2D in China, we found significant associations between plasma levels of citrulline, asparagine, leucine, tryptophan, valine, and most acylcarnitines with changes in GFR (**Supplementary Table 1**). After applying multiple screening criteria (FDR < 0.05, FC > 1.2, VIP > 1), only C5DC and C10 were retained in the comparison between the normal or elevated eGFR and mild reduction eGFR group (NOE vs MR group), while citrulline and 9 acylcarnitines (C2, C4, C5DC, C6, C8, C10, C12, C14:1, and C14-OH) were reserved in the comparison between the normal or elevated eGFR and moderate or severe reduction eGFR group (NOE vs MOMR group). We constructed machine learning models by combining common clinical factors and screened plasma metabolites to predict renal function. The addition of plasma metabolites improved the predictive performance of the XGBoost models for renal function status. Furthermore, we conducted an interpretation of the influence of important metabolites and general clinical features on the progressive impairment of renal function.

Previous studies have shown that plasma amino acids varied significantly in patients with CKD (36, 37). Additionally, changes in plasma valine, glutamate, and glycine have been associated with different stages of CKD (38). Homocysteine and citrulline have been proposed as potential biomarkers for kidney injury and GFR (39). Consistently, our study found that among all amino acids, citrulline exhibited the strongest association with GFR. Citrulline is a non-essential amino acid primarily synthesized in the intestine through the conversion of glutamine (40). In the kidney, citrulline is produced by the enzyme dimethyl arginine dimethyl amino hydrolase (DDAH), which metabolizes asymmetric dimethylarginine (ADMA). Subsequently, citrulline is converted to arginine through the actions of argininosuccinate synthase (ASS) and argininosuccinate lyase (ASL) (41). This finding suggests that renal injury may inhibit the activity of ASS or ASL, leading to abnormal arginine metabolism. Abnormal ADMA metabolism is indicative of arginine metabolism disorders. ADMA possesses biological properties that inhibit nitric oxide (NO) function (42). NO is a potent endothelial vasodilator that maintains vascular tone and regulates blood pressure. We speculate that elevated plasma citrulline levels may be a consequence of the extensive accumulation of ADMA, which inhibits NO synthesis and subsequently leads to decreased GFR.

The strongest correlation with renal function performance was observed with short-chain acylcarnitines. Acylcarnitines are metabolites of fatty acids (FA) that play critical roles in various cellular energy metabolism pathways (43). Acylcarnitines are freely filtered by the glomerulus, with approximately 75% being excreted. Decreased eGFR can result in reduced excretion of acylcarnitines. Acylcarnitines assist in the transport of FA across the inner mitochondrial membrane for β-oxidation (44). Our analysis revealed elevated levels of acylcarnitines in patients with a moderate or severe reduction in eGFR, which may be attributed to a saturated capacity for mitochondrial β-oxidation in the presence of insulin resistance mediated by lipotoxicity, driving the progression of kidney injury (45, 46). Mice fed a high-fat diet exhibited mitochondrial damage in multiple types of kidney cells, possibly due to the inhibition of AMP-activated protein kinase (AMPK) activity, which hinders fatty acid oxidation (FAO) in the kidney (47). Previous research has also demonstrated the significance of short- and medium-chain acylcarnitines as metabolic markers in the progression of renal impairment (13). In our study, both in mild and moderate or severe reduction in eGFR, C5DC, and C10 showed increased levels, indicating their potential as biomarkers for early impaired renal function.

In addition to plasma amino acid and acylcarnitine levels, several other factors such as a long duration of diabetes, high systolic blood pressure (SBP), and high triglyceride (TG) levels are also important risk factors affecting kidney function. Therefore, these groups should receive particular attention, and the frequency of renal function screening should be adjusted accordingly. This will enable more efficient diagnosis and treatment of diseases related to kidney function.

Our study developed an interpretable XGBoost model framework to identify eGFR-related features. By incorporating nine acylcarnitines and citrulline into the model, the AUROC increased significantly from 0.794 to 0.894 (P < 0.001) (**Figure 2**, **Table 2**). The SHAP value of each feature exceeded zero, indicating an increased risk of reduction of eGFR. C5DC > 0.1μmol/L, Cit > 26 μmol/L, triglyceride > 2 mmol/L, age greater > 65 years old and duration of T2D > 10 years were associated with eGFR < 60 mL/min•1.73m² (**Figures 2B–F**). To our knowledge, this is the first use of interpretable machine learning methods to investigate the association of amino acid and acylcarnitine profiles in relation to change in eGFR in Chinese patients with T2D cohort. Another strength of this study is that we used the population of two centers for analysis and the results of the external cohort further proved the reliability of our conclusions. Our prediction model could remind doctors and patients to pay attention to the primary and secondary prevention of renal impairment and increase the renal function screening rate of the high-risk groups.

However, there are limitations in our study. First, proteinuria and glycosylated hemoglobin were not included in the analysis due to too many missing values. However, adjustment for proteinuria in a Japanese cohort study did not abolish the association between amino acid and incident-reduced eGFR (48). Second, the subjects we collected were inpatients with T2D which limits our application to non-hospitalized T2D patients. Third, due to the nature of cross-sectional studies, we cannot prove the existence of causality, which needs to be confirmed in more prospective studies. In the future, we will try to develop models in larger scale data and explore the associations between metabolites with eGFR in prospective study.

Our study demonstrated that plasma metabolites offer new insights into identifying the filtration status of the glomeruli. These metabolites provide information about the cellular metabolic status and function, reflecting the underlying biological processes involved in the onset and progression of diseases. Additionally, analyzing the levels of plasma amino acids and acylcarnitines can help us gain information pertaining to protein metabolism, energy metabolism, and fatty acid metabolism. This information is crucial for the diagnosis, treatment, and monitoring of metabolic disorders, providing valuable clinical insights.

## Data availability statement

This article contains previously unpublished data. Requests to access the datasets should be directed to antf123789@163.com.

## Ethics statement

The studies involving humans were approved by The Ethics Committee for Clinical Research of Liaoning Medical University First Affiliated Hospital and Second Affiliated Hospital of Dalian Medical University. The studies were conducted in accordance with the local legislation and institutional requirements. The human samples used in this study were acquired from primarily isolated as part of your previous study for which ethical approval was obtained. Written informed consent for participation was not required from the participants or the participants’ legal guardians/next of kin in accordance with the national legislation and institutional requirements. Informed consent was waived due to the retrospective character of the cross-sectional study, which is consistent with the Helsinki Declaration.

## Author contributions

TA: Conceptualization, Formal analysis, Methodology, Writing – original draft, Writing – review & editing. ZZ: Writing – original draft. JX: Formal analysis, Writing – original draft. WL: Conceptualization, Methodology, Writing – review & editing. YL: Conceptualization, Methodology, Writing – review & editing. ZF: Funding acquisition, Investigation, Project administration, Resources, Supervision, Writing – review & editing. GZ: Formal analysis, Investigation, Methodology, Supervision, Writing – review & editing.
