# Supplementary material for: Interpretable machine learning identifies metabolites associated with glomerular filtration rate in type 2 diabetes patients
Source: Front Endocrinol (Lausanne). 2024 Jun 10;15:1279034. doi: 10.3389/fendo.2024.1279034 (PMC11194401; doi:10.3389/fendo.2024.1279034)
Supplement: Supplementary file 1 [file DataSheet_1.docx]

Table 1 Amino acids and acylcarnitines analysis in T2D patients.

| NAME | NOE vs MR | | | NOE vs MOSR | | |
| --- | --- | --- | --- | --- | --- | --- |
|  | FDR | FC | VIP | FDR | FC | VIP |
| Ala | 0.56 | 0.98 | 0.56 | 0.03 | 0.90 | 0.44 |
| Arg | 0.89 | 1.01 | 0.38 | 0.01 | 1.30 | 0.88 |
| Asn | 0.01 | 0.93 | 2.05 | <0.001 | 0.87 | 1.19 |
| Asp | 0.50 | 1.03 | 0.12 | 0.53 | 1.04 | 0.34 |
| Cit | <0.001 | 1.17 | 1.27 | <0.001 | 1.56 | 2.03 |
| Cys | 0.50 | 1.03 | 0.25 | 0.01 | 1.20 | 0.84 |
| Gln | 0.23 | 1.06 | 0.98 | 0.12 | 1.10 | 0.83 |
| Glu | 0.40 | 1.03 | 0.47 | 0.61 | 1.02 | 0.46 |
| Gly | 0.48 | 0.97 | 0.73 | 0.35 | 1.05 | 0.46 |
| Hcy | 0.89 | 1.00 | 0.53 | 0.82 | 1.00 | 0.29 |
| His | 0.43 | 1.05 | 0.01 | 0.95 | 1.00 | 0.33 |
| Leu | 0.04 | 0.94 | 1.97 | <0.001 | 0.86 | 1.16 |
| Lys | 0.38 | 1.05 | 0.63 | 0.22 | 1.09 | 0.91 |
| Met | 0.32 | 0.97 | 1.16 | 0.55 | 0.97 | 0.25 |
| Orn | 0.83 | 1.03 | 0.31 | 0.56 | 1.07 | 0.24 |
| Phe | 0.19 | 1.04 | 1.01 | 0.06 | 1.08 | 0.21 |
| Pip | 0.48 | 1.04 | 0.71 | 0.27 | 1.08 | 0.69 |
| Pro | 0.49 | 1.03 | 0.52 | 0.44 | 1.04 | 0.20 |
| Ser | 0.01 | 0.93 | 1.24 | 0.44 | 0.96 | 0.15 |
| Thr | 0.23 | 0.96 | 0.89 | 0.53 | 0.97 | 0.02 |
| Trp | 0.87 | 0.99 | 0.72 | 0.11 | 0.94 | 0.18 |
| Tyr | 0.07 | 0.94 | 1.13 | <0.001 | 0.84 | 0.83 |
| Val | <0.001 | 0.92 | 1.91 | <0.001 | 0.85 | 1.20 |
| C0 | 0.07 | 1.07 | 0.57 | 0.02 | 1.33 | 0.83 |
| C2 | 0.23 | 1.08 | 0.33 | 0.01 | 1.39 | 1.05 |
| C3 | 0.32 | 0.94 | 0.80 | 0.01 | 0.83 | 0.83 |
| C4 | 0.89 | 0.99 | 0.60 | <0.001 | 1.51 | 1.24 |
| C4-OH | 0.76 | 0.84 | 0.59 | 0.48 | 1.46 | 0.69 |
| C4DC | 0.97 | 1.00 | 0.70 | 0.65 | 0.97 | 0.16 |
| C5 | 0.50 | 0.96 | 1.19 | 0.14 | 1.11 | 0.39 |
| C5:1 | 0.59 | 1.03 | 0.50 | <0.001 | 1.25 | 0.73 |
| C5-OH | 0.03 | 0.91 | 1.58 | 0.03 | 0.88 | 0.88 |
| C5DC | <0.001 | 1.27 | 1.10 | <0.001 | 2.53 | 2.50 |
| C6 | 0.11 | 1.08 | 1.11 | <0.001 | 1.63 | 1.83 |
| C8 | <0.001 | 1.20 | 1.73 | <0.001 | 1.81 | 2.24 |
| C10 | <0.001 | 1.23 | 1.63 | <0.001 | 1.84 | 2.14 |
| C12 | <0.001 | 1.17 | 1.42 | <0.001 | 1.49 | 1.64 |
| C14 | 0.07 | 1.08 | 0.68 | <0.001 | 1.26 | 0.90 |
| C14:1 | <0.001 | 1.20 | 1.18 | <0.001 | 1.71 | 2.04 |
| C14-OH | 0.40 | 1.06 | 0.13 | <0.001 | 1.42 | 1.26 |
| C14DC | 0.03 | 0.87 | 1.16 | 0.14 | 1.16 | 0.60 |
| C16 | 0.92 | 1.00 | 0.20 | 0.70 | 0.98 | 0.20 |
| C16-OH | 0.73 | 1.03 | 0.07 | 0.47 | 1.09 | 0.09 |
| C16:1-OH | 0.31 | 1.05 | 0.18 | 0.56 | 1.03 | 0.00 |
| C18 | 0.43 | 1.03 | 0.00 | 0.09 | 1.09 | 0.02 |
| C20 | 0.50 | 0.96 | 1.05 | 0.14 | 1.14 | 0.20 |
| C22 | 0.11 | 0.92 | 1.32 | 0.56 | 1.04 | 0.08 |
| C24 | 0.11 | 0.92 | 1.22 | 0.64 | 0.97 | 0.37 |
| C26 | 0.23 | 0.94 | 1.11 | 0.34 | 0.94 | 0.54 |

*NOE, Normal renal function; MR, compensation period of renal function; MOSR, Renal insufficiency.*

*FDR, Benjaminiand-Hochberg false discovery rate; FC, fold change; VIP, the metabolites were listed in a decreasing order based on variable importance in the projection values.*

*Ala, alanine; Arg, argnine; Asn, asparagine; Asp, aspartic acid; Cit, citrulline; Cys, cysteine; Gln, glutamine; Glu,* *glutamic acid; Gly, glycine; Hcy, homocysteine; His, hlstidine; Leu, leucine; Lys, lysine; Met, methionine; Orn, ornithine; Phe, phenylalanine; Pip, Piperamide; Pro, proline; Ser, serine; Thr, threonine; Trp, tryptophane; Tyr, threonine; Val, valine. C0, free carnitine; C2, acetylcarnitine; C3, propionylcarnitine; C4, butyrylcarnitine; C4-OH, hydroxylbutyrylcarnitine; C4DC, succinylcarnitine; C5, isovalerylcarnitine; C5:1, tiglylcarnitine; C5-OH, 3-hydroxyisovalerylcarnitine; C5DC, glutarylcarnitine; C6, hexanoylcarnitine; C8, octanoylcarnitine; C10, decanoylcarnitine; C12, lauroylcarnitine; C14, myristoylcarnitine; C14:1, tetradecenoylcarnitine; C14-OH, 3-hydroxyl-tetradecanoylcarnitine; C14DC, tetradecanoyldiacylcarnitine; C16, palmitoylcarnitine; C16-OH, 3-hydroxypalmitoylcarnitine; C16:1-OH, 3-hydroxypalmitoleylcarnitine; C18, octadecanoylcarnitine; C20, arachidic carnitine; C22, behenic carnitine; C24, tetracosanoic carnitine; C26, hexacosanoic carnitine.*

**C**

**A**


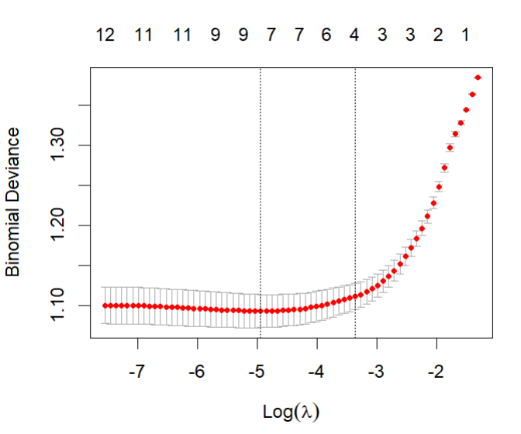

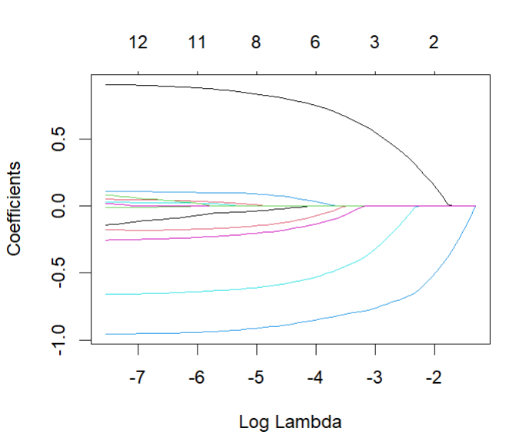


NOE vs MR


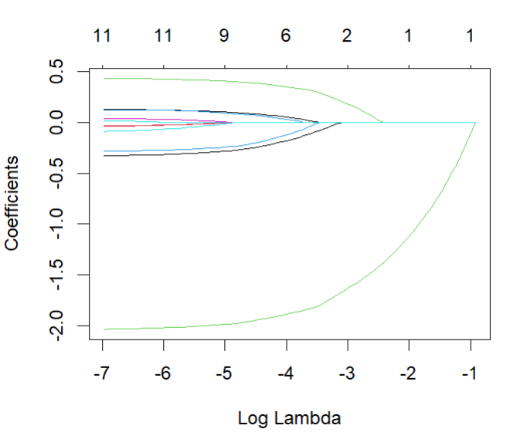

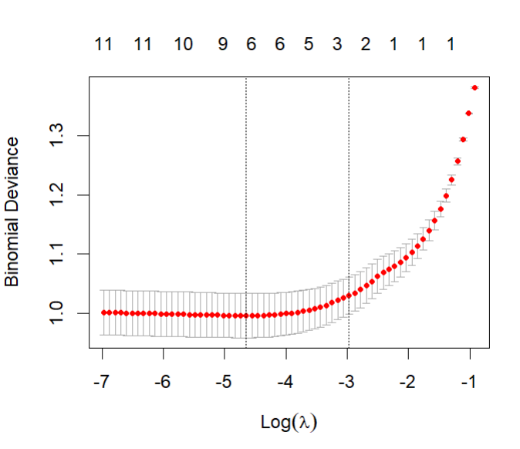


NOE vs MOSR

**B**

**D**

Fig. 1 Identify the predicted clinical factors via LASSO. (A, C) Lasso coefficients profiles of the 12 clinical factors for NOE vs MR and NOE vs MOSR, respectively. (B, D) The optimal tuning parameters (lambda) in the LASSO analysis were selected with 10-fold cross-validation and one standard error rule. Dashed vertical lines were drawn at the log of the optimal values of lambda by using the minimum criteria and the 1SE criteria.

*NOE, Normal or elevated eGFR; MR, Mild reduction eGFR; MOMR, moderate or severe reduction eGFR.*

**A**


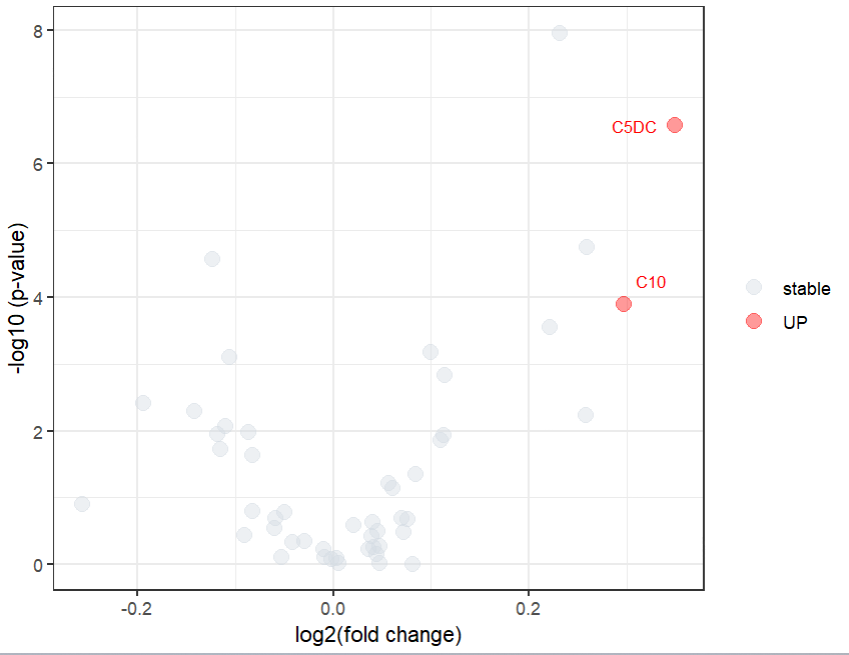


NOE vs MR

NOE vs MOSR


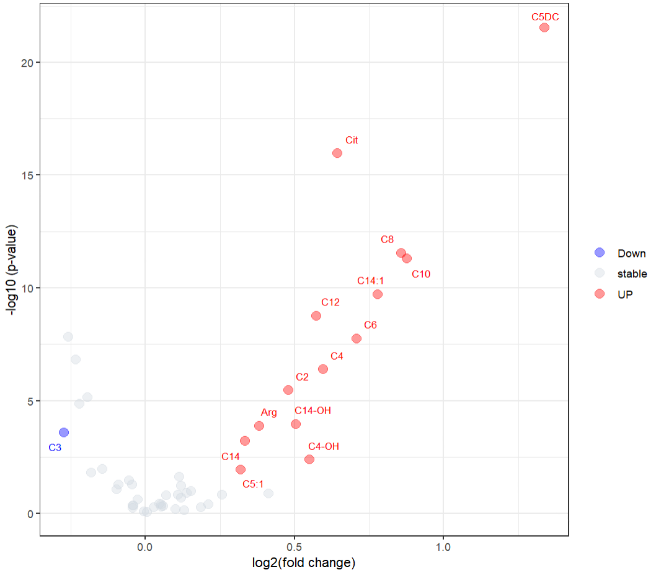


**B**

C

Fig. 2 Volcano plots of the plasma metabolites. (A) was a comparison of NOE and MR and (B) was a comparison of NOE and MOMR. The x-axis is log2 fold change and the y-axis represents the minus log10 p-value. Over-expressed and under-expressed plasma metabolites were marked with red and blue colors, respectively.

*NOE, Normal or elevated eGFR; MR, Mild reduction eGFR; MOMR, moderate or severe reduction eGFR.*

*Arg, argnine; Cit, citrulline; C2, acetylcarnitine; C3, propionylcarnitine; C4, butyrylcarnitine; C4-OH, 3-hydroxy butyrylcarnitine; C5:1, tiglylcarnitine; C5DC, glutarylcarnitine*; *C6, hexanoylcarnitine; C8, octanoylcarnitine; C10, decanoylcarnitine; C12, lauroylcarnitine; C14, myristoylcarnitine; C14:1, tetradecenoylcarnitine; C14-OH, 3-hydroxyl-tetradecanoylcarnitine.*

**A**


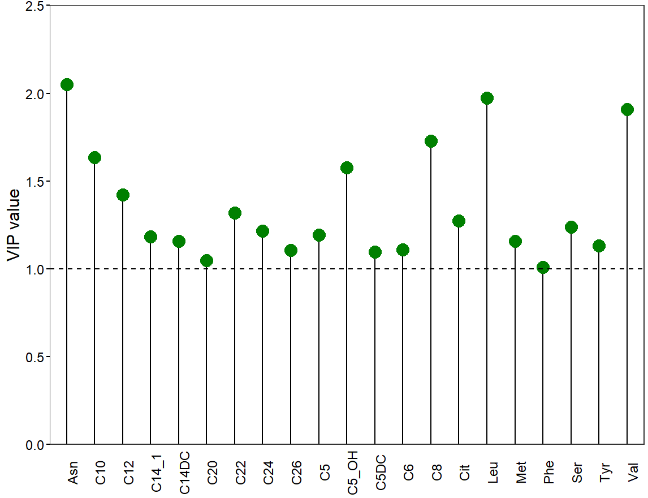


NOE vs MR NOE vs MOSR


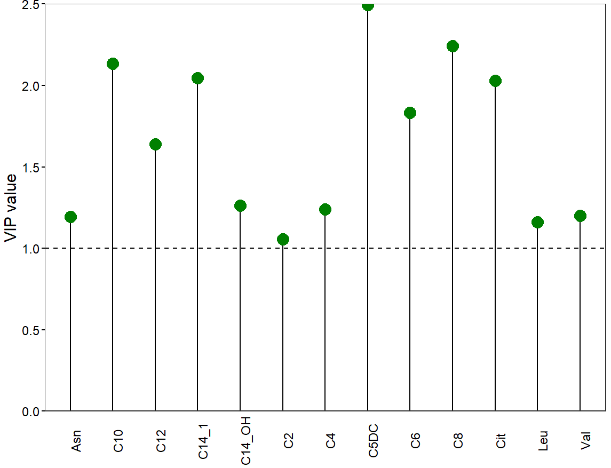


**B**

Fig. 3 VIP values of plasma metabolites on renal function. (A) was a comparison of NOE vs MR and (B) was a comparison of NOE vs MOMR. Only metabolites with VIP value>1 were represented.

*VIP, variable importance in the projection values; NOE, Normal or elevated eGFR; MR, Mild reduction eGFR; MOMR, moderate or severe reduction eGFR; Asn, asparagine; Cit, citrulline; Leu, leucine; Met, methionine; Phe, phenylalanine; Ser, tryptophan, Tyr, tyrosine; Val, valine; C2, acetylcarnitine; C4, butyrylcarnitine; C5, isovalerylcarnitine; C5-OH, 3-hydroxyisovalerylcarnitine; C5DC, glutarylcarnitine*; *C6, hexanoylcarnitine; C8, octanoylcarnitine; C10, decanoylcarnitine; C12, lauroylcarnitine; C14:1, tetradecenoylcarnitine; C14-OH, 3-hydroxyl-tetradecanoylcarnitine*

Table 2 Performance of predictive models for NOE vs MR in the internal validation cohort

| Model | AUROC | p^a^ | AUPRC | P^b^ |
| --- | --- | --- | --- | --- |
| LR1 | 0.801(0.75-0.85) | Ref | 0.534(0.42-0.64) | Ref |
| SVM1 | 0.792(0.74-0.84) | 0.28 | 0.538(0.43-0.64) | 0.65 |
| RF1 | 0.775(0.71-0.83) | 0.13 | 0.501(0.39-0.62) | 0.06 |
| XGBoost1 | 0.799(0.74-0.85) | 0.77 | **0.561(0.45-0.66)** | 0.006 |
| LR2 | 0.797(0.74-0.84) | Ref | 0.524(0.42-0.63) | Ref |
| SVM2 | 0.793(0.74-0.84) | 0.69 | 0.540(0.43-0.64) | 0.11 |
| RF2 | 0.758(0.70-0.82) | 0.03 | 0.512(0.39-0.62) | 0.52 |
| XGBoost2 | 0.784(0.73-0.84) | 0.36 | 0.544(0.43-0.65) | 0.16 |

*AUROC, the area under curve of the receiver operating characteristic curve; AUPRC, area under the precision recall curve; Ref, reference.*

*LR1, SVM1, RF1, XGBoost1 are models that only includes traditional clinical factors; LR2, SVM2, RF2, XGBoost2 add plasma metabolites.*

*a Delong test for area under the curve of receiver operating characteristic curve.*

*b Delong test for area under the curve of precision-recall curve.*

*NOE, normal renal function; MR, compensation period of renal function.*

*LR, logistic regression; SVM, support vector machine; RF, random forest; XGBoost, eXtreme Gradient Boosting.*

Fig. 4 Performance of predictive models for NOE vs MR in the internal validation cohort. (A, C) Receiver operating characteristic curves of models with clinical factors and the combination of plasma metabolites and clinical factors, respectively; (B, D) Precision recall curves of models with clinical factors and the combination of plasma metabolites and clinical factors, respectively.

C

A


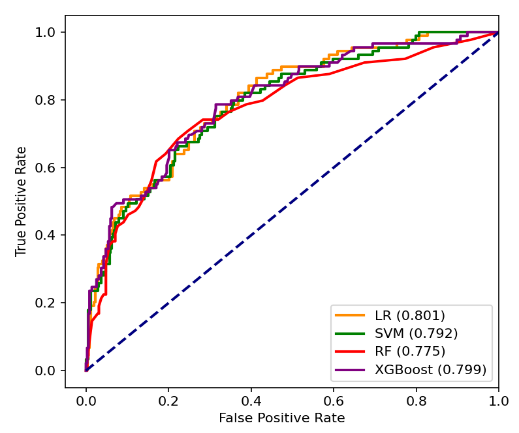

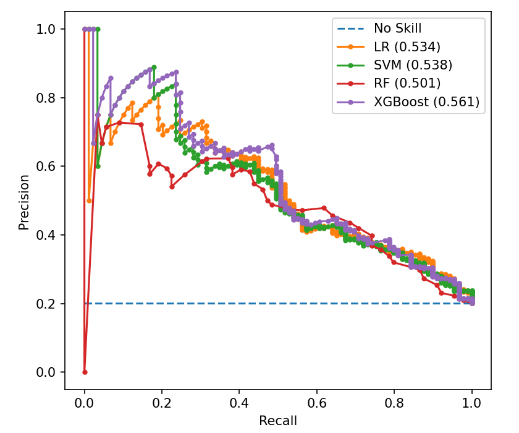

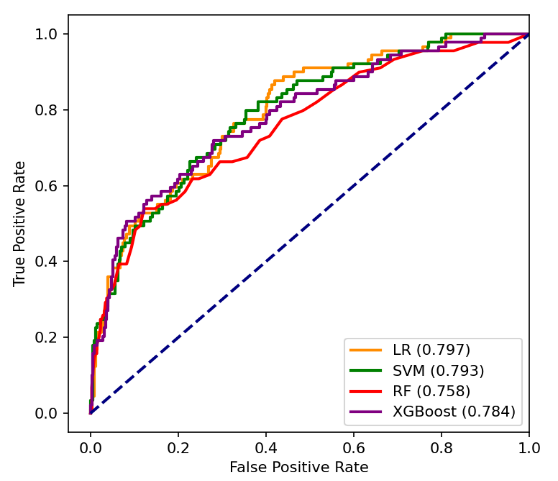

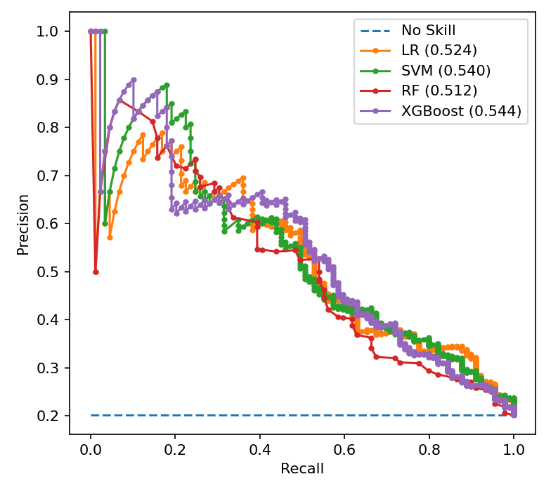


B

D

*LR, logistic regression; SVM, support vector machine; RF, random forest; XGBoost, eXtreme Gradient Boosting; No skill is the reference line.*

Table 3 Performance of predictive models for NOE vs MOSR in the internal validation cohort

| Model | AUROC | p^a^ | AUPRC | P^b^ |
| --- | --- | --- | --- | --- |
| LR1 | 0.784(0.71-0.84) | Ref | 0.341(0.21-0.48) | Ref |
| SVM1 | 0.718(0.63-0.80) | 0.04 | 0.357(0.23-0.51) | 0.65 |
| RF1 | 0.765(0.69-0.84) | 0.49 | 0.384(0.24-0.51) | 0.14 |
| XGBoost1 | 0.794(0.73-0.85) | 0.59 | 0.374(0.24-0.53) | 0.07 |
| LR2 | 0.836(0.78-0.90) | Ref | 0.527(0.37-0.66) | Ref |
| SVM2 | 0.836(0.77-0.90) | 0.98 | 0.489(0.33-0.65) | 0.07 |
| RF2 | 0.881(0.82-0.93) | 0.08 | 0.622(0.48-0.75) | <0.001 |
| XGBoost2 | **0.894(0.85-0.94)** | 0.02 | **0.648(0.50-0.77)** | <0.001 |

*AUROC, the area under curve of the receiver operating characteristic curve; AUPRC, area under the precision recall curve; Ref, reference.*

*LR1, SVM1, RF1, XGBoost1 are models that only includes traditional clinical factors; LR2, SVM2, RF2, XGBoost2 add plasma metabolites.*

*a Delong test for area under the curve of receiver operating characteristic curve.*

*b Delong test for area under the curve of precision-recall curve.*

*LR, logistic regression; SVM, support vector machine; RF, random forest; XGBoost, eXtreme Gradient Boosting.*


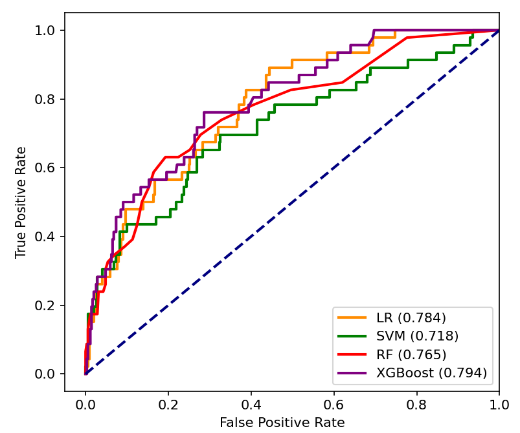

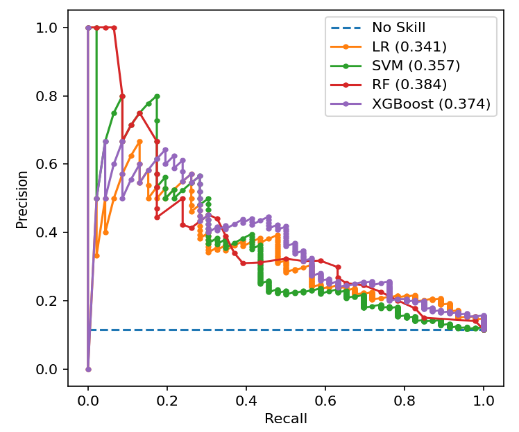


**A**

**B**

**C D**


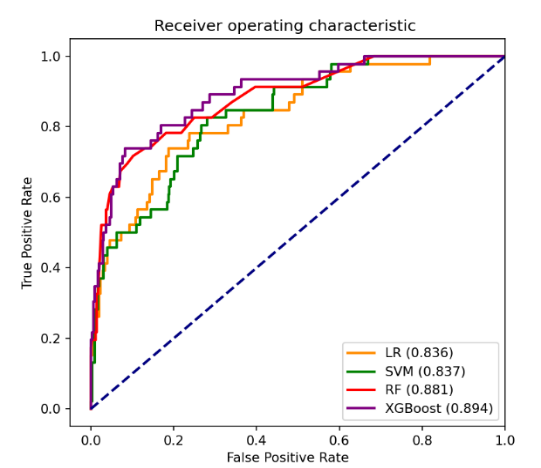

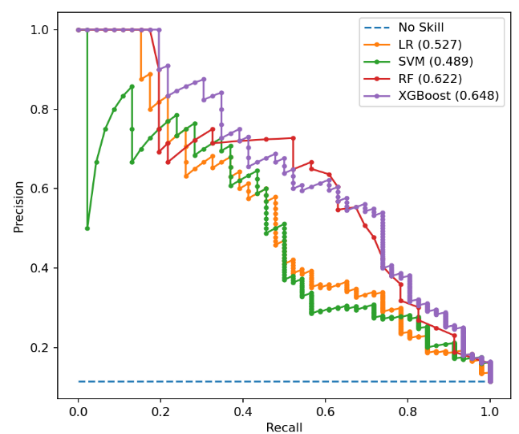


Fig. 5 Performance of predictive models for NOE vs MOSR in the internal validation cohort. (A, C) Receiver operating characteristic curves of models with clinical factors and the combination of plasma metabolites and clinical factors, respectively; (B, D) Precision recall curves of models with clinical factors and the combination of plasma metabolites and clinical factors, respectively.

*LR, logistic regression; SVM, support vector machine; RF, random forest; XGBoost, EXtreme Gradient Boosting; No skill is the reference line.*


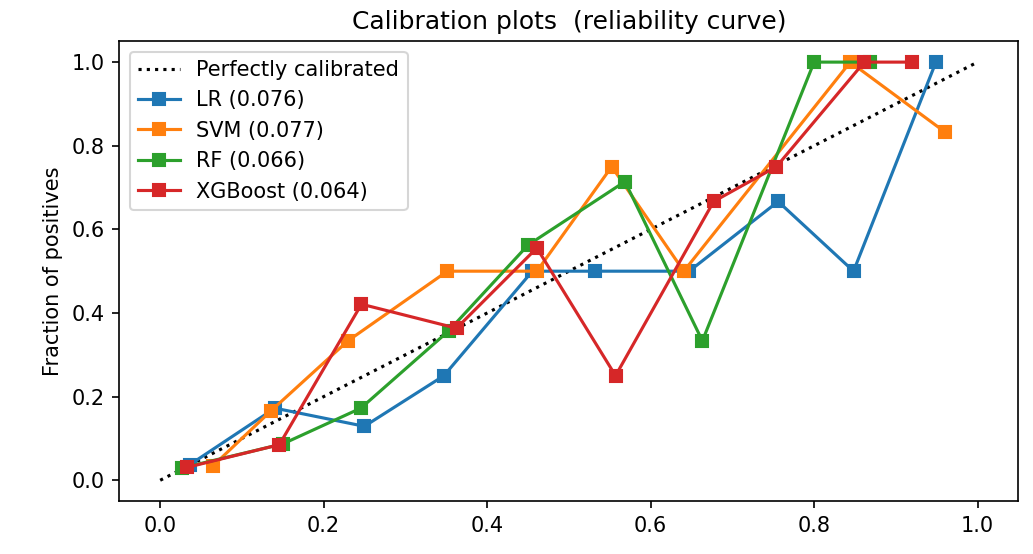


Fig. 6 The calibration curves of predictive models based on a combination of plasma metabolites and clinical factors for NOE vs MOSR in the internal validation cohort.

*The values in brackets represent the Brier score of the corresponding prediction model.*

*Perfectly calibrated is the reference line.*

*LR, logistic regression; SVM, support vector machine; RF, random forest; XGBoost, eXtreme Gradient Boosting.*

A


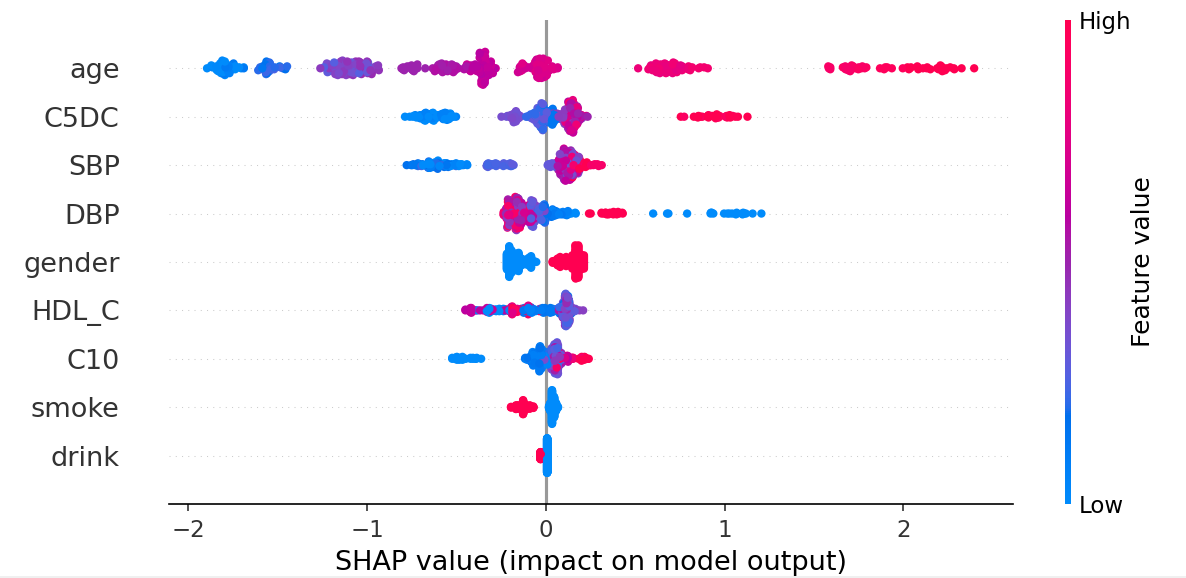


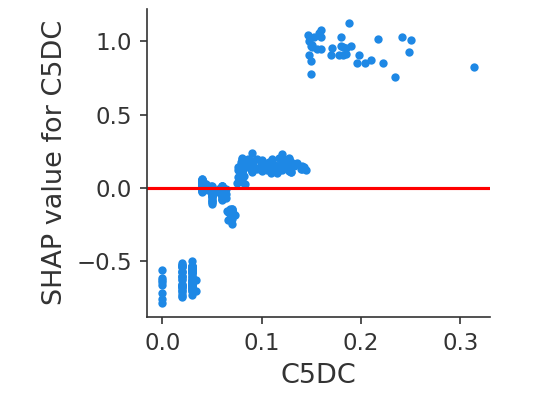

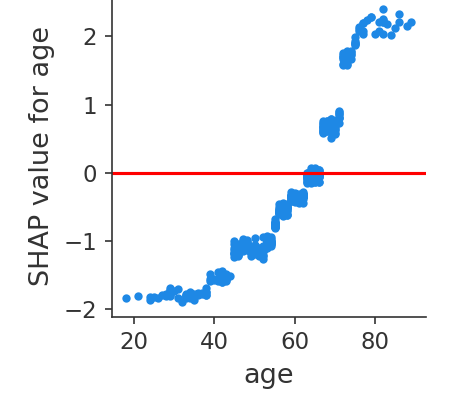


B

C

Fig. 7 SHAP plot of XGBoost2 model of NOE vs MR. (A) SHAP summary plot. Features are ranked from top to bottom according to their importance. Each dot on the plot is a SHAP value for each feature. Red dots indicate high feature values, but blue dots represent low feature values for the per-patient model. (B-C) SHAP dependence plot. The SHAP value of each feature exceeded zero, indicating an increased risk of renal function compensation.

*SHAP, Shapley Additive explanation*

*SBP, systolic blood pressure; DBP,* *diastolic blood pressure; HDL:C, high-density lipoprotein cholesterol; C5DC, glutarylcarnitine; C10, decanoylcarnitine.*
